# Retraction: Panax notoginseng saponins regulate VEGF to suppress esophageal squamous cell carcinoma progression *via* DVL3-mediated Wnt/β-catenin signaling

**DOI:** 10.1039/d1ra90072b

**Published:** 2021-02-03

**Authors:** Laura Fisher

**Affiliations:** Royal Society of Chemistry Thomas Graham House, Science Park, Milton Road Cambridge CB4 0WF UK advances-rsc@rsc.org

## Abstract

Retraction of ‘Panax notoginseng saponins regulate VEGF to suppress esophageal squamous cell carcinoma progression *via* DVL3-mediated Wnt/β-catenin signaling’ by Xiaoqi Chen *et al.*, *RSC Adv.*, 2020, **10**, 3256–3265, DOI: 10.1039/C9RA07830D.

The Royal Society of Chemistry hereby wholly retracts this *RSC Advances* article due to concerns with the reliability of the data. The images in the article, and the raw data provided by the authors, were screened by an image integrity expert.

The published western blots in Fig. 3A (VEGF panel), Fig. 4F (VEGF panel) and Fig. 7A (DVL3 and β-catenin panels) show signs of manipulation and cloning in the backgrounds of the images. In addition, while the individual blots in the raw data provided by the authors matched the figures, the backgrounds did not match the figure panels. Therefore, the raw data provided by the authors cannot be used to validate the published data.

Given the significance of the concerns about the validity of both the data in the article and the raw data provided by the authors, the findings presented in this paper are not reliable.

The authors agree to the retraction.

Signed: Laura Fisher, Executive Editor, *RSC Advances*.

Date: 19^th^ January 2021.

## Supplementary Material

